# Measuring health-related quality of life in sarcopenia: summary of the SarQoL psychometric properties

**DOI:** 10.1007/s40520-023-02438-3

**Published:** 2023-05-23

**Authors:** Charlotte Beaudart, Jean-Yves Reginster, Jotheeswaran Amuthavalli Thiyagarajan, Ivan Bautmans, Jürgen Bauer, Nansa Burlet, Matteo Cesari, Antonio Cherubini, Cyrus Cooper, Alfonso J. Cruz-Jentoft, Bess Dawson-Hughes, Roger A. Fielding, Nicholas C. Harvey, Francesco Landi, Andrea Laslop, Stefania Maggi, Beatriz Montero-Errasquin, Prieto Yerro María Concepción, Yves Rolland, René Rizzoli, Marjolein Visser, Olivier Bruyère

**Affiliations:** 1 Division of Public Health, Epidemiology and Health Economics, WHO Collaborating Center for Public Health Aspects of Musculo-Skeletal Health and Ageing, University of Liège, Liège, Belgium; 2 Department of Ageing and Life Course, WHO, Geneve, Switzerland; 3 Frailty in Ageing Research Department, Vrije Universiteit Brussel, Brussels, Belgium; 4 Center for Geriatric Medicine and Network Aging Research (NAR), Heidelberg University, Heidelberg, Germany; 5 Department of Clinical Sciences and Community Health, University of Milan, Milan, Italy; 6 Geriatric Unit, IRCCS Istituti Clinici Scientifici Maugeri, Milan, Italy; 7 Geriatria, Accettazione geriatrica e Centro di ricerca per l’invecchiamento, IRCCS INRCA, Ancona, Italy; 8 MRC Lifecourse Epidemiology Centre, University of Southampton, Southampton, UK; 9 Servicio de Geriatría. Hospital Universitario Ramón y Cajal (IRYCIS), Madrid, Spain; 10 Jean Mayer USDA Human Nutrition Research Center on Aging, Tufts University, Boston, MA USA; 11 Nutrition, Exercise Physiology and Sarcopenia Laboratory, Jean Mayer USDA Human Nutrition Research Center on Aging, Tufts University, Boston, USA; 12 NIHR Southampton Biomedical Research Centre, University of Southampton and University Hospital Southampton NHS Foundation Trust, Southampton, UK; 13 Fondazione Policlinico Universitario “Agostino Gemelli” IRCCS, 00168 Rome, Italy; 14 Scientific Office, Austrian Medicines and Medical Devices Agency, Vienna, Austria; 15 CNR Aging Branch-IN, Padua, Italy; 16 Agencia Española de Medicamentos y Productos Sanitarios, Madrid, Spain; 17 Gérontopôle de Toulouse, Institut du Vieillissement, Centre Hospitalo-Universitaire de Toulouse, CERPOP UMR 1295, University of Toulouse III, Inserm, Toulouse, France; 18 Service of Bone Diseases, Faculty of Medicine, Geneva University Hospitals, Geneva, Switzerland; 19 Department of Health Sciences, Faculty of Science, Vrije Universiteit Amsterdam and the Amsterdam Public Health Research Institute, Amsterdam, The Netherlands

**Keywords:** Sarcopenia, Quality of life, Psychometric properties, Validation, HRQoL

## Abstract

Patient perspectives are now widely recognized as a key element in the evaluation of health interventions. Therefore, the provision of specific and validated Patient Reported Outcome Measures that emphasize the lived experience of patients suffering from specific diseases is very important. In the field of sarcopenia, the only validated specific health-related quality of life (HRQoL) instrument available is the *Sarcopenia Quality of Life* questionnaire (SarQoL). This self-administrated HRQoL questionnaire, developed in 2015, consists of 55 items arranged into 22 questions and has currently been translated into 35 languages. Nineteen validation studies performed on SarQoL have consensually confirmed the capacity of SarQoL to detect difference in HRQoL between older people with and without sarcopenia, its reliability and its validity. Two further observational studies have also indicated its responsiveness to change. A short form SarQoL, including only 14 items has further been developed and validated to reduce the potential burden of administration. Research on the psychometric properties of SarQoL questionnaire is still encouraged as the responsiveness to change of SarQoL has not yet been measured in the context of interventional studies, as limited prospective data currently exist and as there is still not cut-off score to define a low HRQoL. In addition, SarQoL has mainly been used in community-dwelling older individuals with sarcopenia and would benefit to be studied in other types of populations. This review aims to provide to researchers, clinicians, regulators, pharmaceutical industries and other stakeholders a clear summary of comprehensive evidence on the SarQoL questionnaire published up to January 2023Query.

**Supplementary Information:**

The online version contains supplementary material available at 10.1007/s40520-023-02438-3.

## Introduction

Patient-reported outcome measures (PROMs) aim to assess patients' experiences, such as pain, quality of life or satisfaction with care. There is an increasing emphasis on patient-centered research, and patient perspectives are now recognized as a critical element in the evaluation of health interventions. Indeed, using a PROMs will allow essential aspects of patient-relevant treatment effectiveness to be captured. Government regulatory agencies such as the Food and Drug Administration (FDA) or the European Medicines Agency (EMA) have encouraged the appropriate use of PROMs in regulatory studies [1, 2]. Health-related quality of life (HRQoL), one of the most commonly measured PROMs, can be measured using generic HRQoL questionnaires, such as the SF-36 or the EQ-5D, as well as specific instruments. As their category name suggests, disease-specific HRQoL questionnaires tend to measure more specific elements of the disease in question, can detect subtle effects of a disease on HRQoL, and are therefore theoretically more sensitive to treatment-related changes than generic HRQoL measures [3]. Because of the characteristics offered by specific instruments, many disease-specific HRQoL have been developed in the past few years such as the diabetes-specific quality of life questionnaire (DMQoL), the osteoarthritis knee and hip quality of life questionnaire (OAKHQOL) and the rheumatoid arthritis quality of life questionnaire (RAQoL).

According to the 2nd European Working Group on Sarcopenia in Older People (EWGSOP2) [4], sarcopenia can be defined as a “progressive and generalized skeletal muscle disorder that is associated with increased likelihood of adverse outcomes including falls, fractures, physical disability and mortality”. Some recent meta-research syntheses have highlighted the increased likelihood of functional decline, falls, fractures, hospitalizations and even death in individuals with sarcopenia [5–8]. While these investigations have mainly focused on so-called “hard clinical outcomes”, there has also been a growing interest in the lived experience of people with sarcopenia. Until 2015, HRQoL in individuals with sarcopenia was only measured using generic instruments. Some studies reported reduced HRQoL in individuals with sarcopenia, but this was mainly observed in particular domains of HRQoL, mainly related to the physical function and mobility of individuals [9–12]. These results suggested that quality of life of people with sarcopenia may be affected in specific domains that are directly related to the disease and therefore to muscle function. To complement the information obtained from these generic tools and to obtain a more specific measurement of HRQoL in this population, a group of experts decided in 2015 to develop the first sarcopenia-specific HRQoL questionnaire, namely the Sarcopenia & Quality of Life (SarQoL) questionnaire [13]. As one year later, in 2016, the 10th version of International Classification of Diseases (ICD-10-CM codes) added a code for the diagnosis of sarcopenia[14], the availability of a specific HRQoL assessment for sarcopenia is particularly interesting. To date, the SarQoL questionnaire (http://www.sarqol.org) is the only sarcopenia-specific validated HRQoL instrument available for older people in the scientific literature [13, 15]. Eight years after its development, SarQoL is being used in many epidemiological and interventional studies worldwide.

This narrative review aims to provide an update on the characteristics and validated implementation of the SarQoL questionnaire, relevant to researchers, clinicians, regulators, pharmaceutical industry and other stakeholders. Throughout a in depth literature research in Scopus and Medline bibliographic databases, the SarQoL-related scientific literature published up to January 2023 is presented in this review.

## Development of the SarQol questionnaire

The Sarcopenia Quality of Life (SarQoL) self-administered questionnaire was developed in 2015 by a research team of thirteen French-speaking experts from Belgium, France and Switzerland. This team of experts comprised geriatricians, rheumatologists, specialists in physical medicine and rehabilitation, researchers in the field of sarcopenia, French linguists, experts in questionnaire methodology and statisticians. The questionnaire was developed in four steps, based on scientific guidelines and the literature available at the time of the development of quality-of-life instruments [16, 17]. In the first step (i.e., item generation), a systematic literature review and interviews with five individuals with sarcopenia (diagnosed according to EWGSOP1 criteria [18]) and seven experts in the field were conducted to generate a list of items related to HRQoL in sarcopenia. This initial list was composed of 180 items, which was considered too extensive to develop a questionnaire to be completed by a population of older adults. Therefore, a second step, item reduction, was undertaken to reduce this list to the most relevant items to be included in a PROM. Twenty-one individuals with sarcopenia and seven experts in the field of sarcopenia were invited to participate in this item reduction phase and were asked to select the items they considered most relevant from the 180 proposed items. Using a cut-off point of 0.5 (frequency x importance) and expert consensus, a final list of 55 items was obtained, divided into seven domains. The list of items was then divided into 22 questions by the expert panel. Finally, the questionnaire was pre-tested on a sample of 20 older individuals with sarcopenia who were asked about the relevance and comprehensiveness of each question.

The final SarQoL questionnaire, therefore, consists of 55 items structured into seven domains of HRQoL and composed of 22 questions rated on a 3-, 4- and 5-point Likert scale of frequency and intensity (the English version of SarQoL is available in the Appendix). The seven domains of HRQoL are Physical and Mental Health, Locomotion, Body Composition, Functionality, Activities of Daily Living, Leisure Activities and Fears. The total score of the SarQoL questionnaire ranges from 0 to 100, and individual scores can also be generated for each domain (the SarQoL scoring system can be obtained by contacting the lead authors at info@sarqol.org). A lower score indicates a lower QoL. The questionnaire can be used free of charge for all academic or clinic unsponsored studies.

## Validated trenslations of SarQol

So far, SarQoL is available in 35 different languages. Leading SarQoL developers were contacted before each translation and provided instructions based on Beaton’s recommendations [19] and COSMIN guidelines [20]. From those 35 translated versions, 19 have currently been validated in a population of individuals with sarcopenia. The following versions of SarQoL have been validated: Brazilian [21], Chinese [22], Dutch [23], English [24], French [15], Greek [25], Hungarian [26], Korean [27], Lithuanian [28], Persian [29], Polish [30], Romanian [31], Russian [32], Serbian [33], Spanish [34, 35], Taiwanese [36], Turkish [37] and Ukrainian [38].

## Psychometric properties of SarQol

The psychometric properties of SarQoL have been measured in 24 different populations; 19 validation studies (number of participants = 3657 of whom 776 (21.2%) were sarcopenic) and five studies aimed at assessing specific psychometric properties (number of participants = 1150) [39–43].

In 2022, an independent systematic literature review was published to analyse the structural characteristics and psychometric properties of SarQoL in different languages [44]. At that time, the authors were able to include 14 individual studies reporting on the psychometric properties of SarQoL. Surprisingly, they did not report the psychometric properties of the French version. Moreover, the Hungarian, Brazilian, Taiwanese and Persian versions of SarQoL were published afterwards.

In these different publications on the psychometric properties of SarQoL, the discriminant validity, reliability (i.e., internal consistency, test–retest reliability, measurement error), validity (i.e., content validity, construct validity), responsiveness and floor/ceiling effects of SarQoL were reported (Table 1).

**Table 1:** Studies supporting psychometric validation of SarQoL

Reference	Translation/ other validation	Population: N, Sex, Age	Prevalence of sarcopenia	Reliability			Validity		Responsiveness	Floor / Ceiling effects
				Internal consistency (Cronbach’s alpha)	Test–retest reliability (ICC (95%CI))	Measurement error (SEM, SDC)	Content validity¶	Convergent/divergent validity		
Alekna 2019 [28]	Translation: Lithuanian	n = 176; F: n = 105, M: n = 71 Age: median = 78.2; range = 74.1–82.6	EWGSOP2 criteria: n = 58	0.95	0.98 (0.96–0.99)	SEM = 0.18 SDC = 0.49	NA, but pre-test performed (n = 16)	SF-36, EQ5D Consistent construct validity (convergent and divergent)	NA	None
Beaudart 2017 [15]	French (original version)	n = 296; F: n = 169, M: n = 127 Age: mean = 73.3; range = 68.9–78.6	EWGSOP criteria: n = 43	0.87	0.91 (0.82–0.95)	SEM = 4.06 SDC = 11.34	NA, but pretest performed (n = 20)	SF-36, EQ5D Consistent construct validity (convergent and divergent)	NA	None
Beaudart 2017 [24]	Translation: English	n = 297; F: n = 137, M: n = 160 Age: mean = 79.5 ± 2.62	EWGSOP criteria: n = 14	0.88	0.95 (0.92–0.97)	SEM = 4.20 SDC = 11.65	NA, but pretest performed (n = 10)	SF-36, EQ5D Consistent construct validity (convergent and divergent)	NA	None
De Souza Orlandi 2022 [21]	Translation: Brazil	N = 221; F: n = 151, M: n = 70 Age: mean = 70 years	EWGSOP2 criteria: n = 55	0.98	0.98 (0.90–0.99)	SEM = 2.17 SDC = 6‡	NA	SF-36, EQ5D Consistent construct validity (convergent)	NA	None
Dzhus 2020 [38]	Translation: Ukranian	n = 49; F: n = 20, M: n = 29 Age: median = 71; range = 67.0–75.5	Ishii test: n = 28	0.90	0.99 (0.99–0.99)	NA	NA, but pretest performed (n = 10)	SF-36, EQ5D Consistent construct validity (convergent)	NA	None
Erdogan 2021 [37]	Translation: Turkish	n = 100; F: n = 71, M: n = 29 Age: mean = 74.7 ± 6.1	EWGSOP2 criteria: n = 9	0.88	0.97 (0.94–0.98)	NA	NA, but pretest performed (n = 10)	SF-36, EQ5D Consistent construct validity (convergent and divergent)	NA	None
Fabrega-Cuadros 2020 [35]	Translation: Spanish	n = 252; F: n = 208, M: n = 44 Age: median = 74.0; range = 70.0–78.0	EWGSOP2 criteria: n = 66	0.90	0.99 (0.98–0.99)	NA	NA	SF-36, EQ5D Consistent construct validity (convergent and divergent)	NA	None
Gasparik 2017 [31]	Translation: Romanian	n = 100; F: n = 69, M: n = 31 Age: median = 72; range = 67–79	EWGSOP criteria: n = 13	0.95	NA	NA	NA	SF-36, EQ5D Consistent construct validity (convergent and divergent)	NA	None
Geerinck 2018 [23]	Translation: Dutch	n = 92; F: n = 40, M: n = 52 Age: median = 82; range = 73–85	EWGSOP criteria: n = 30	0.88	0.98 (0.95–0.99)	SEM = 2.54 SDC = 7.05	NA, but pretest performed (n = 14)	SF-36, EQ5D Consistent construct validity (convergent and divergent)	NA	None
Geerinck 2022 [26]	Translation: Hungarian	N = 70, F = 54, M = 16 Age: median = 80, range 68.5–82.5	EWGSOP criteria: n = 0 Low grip strength (probable sarcopenia) n = 30	0.89	NA	NA	NA, but pretest performed (n = 40)	SF-36, EQVAS and EQ5D Consistent construct validity (convergent and divergent)	NA	None
Konstantynowicz 2018 [30]	Translation: Polish	n = 106; F: n = 69, M: n = 37 Age: Mean = 73.3 ± 5.94	EWGSOP criteria: n = 60	0.92	0.99 (0.99–0.99)	SEM = 1.07 SDC = 2.96	NA, but pretest performed (n = 10)	SF-36, EQ5D Consistent construct validity (convergent)	NA	None
Le 2021 [22]	Translation: Chinese	n = 159; F: n = 74, M: n = 85 Age: NR	AWGS criteria: n = 51	0.87	0.99 (0.99–0.99)	NA	NA, but pretest performed (n = 10)	SF-36, EQ5D Consistent construct validity (convergent and divergent)	NA	None
Lee 2022 [36]	Translation: Taiwanese	n = 100; F: n = 72; M: n = 18 Age only reported by categories (> 65 years)	N = 50	0.85	0.97 (0.95–0.98)	NA	NA, but pretest performed (n = 10)	SF-12, EQ5D Consistent construct validity (convergent and divergent)	NA	None
Mahmoodi 2022 [29]	Translation: Persian	N = 128; F: n = 53, M: n = 75 Age: mean = 74.8 (SD 5.05)	AWGS criteria: n = 88	0.88	0.99 (0.99–0.99)	NA	CVR = 0.8–1 and The CVI = 1	SF-36, EQ5D Consistent construct validity (convergent and divergent)	NA	None
Matijevic 2020 [33]	Translation: Serbian	N = 699: F = 508, M = 191 Age: median = 70, range = 67–74	EWGSOP2 criteria: n = 12	0.87	NA	NA	NA	SF-36, EQ5D Consistent construct validity (convergent and divergent)	NA	None
Montero-Errasquin 2022 [34]	Translation: Spanish	n = 86; F: n = 69, M: n = 17 Age: median = 77; range = 70–91	EWGSOP criteria: n = 16; FNIH criteria n = 13	0.84	0.97 (0.92–0.99)	SEM = 1.73 SDC = 4.81	NA, but pretest performed (n = 10)	SF-36, EQ5D Consistent construct validity (convergent and divergent)	NA	None
Safonova 2019 [32]	Translation: Russian	N = 100 Age: mean = 74 ± 6.5	EWGSOP criteria: n = 50	0.92	0.93 (0.91–0.96)	NA	NA	SF-36, EQ5D Consistent construct validity (convergent)	NA	None
Tsekoura 2020 [25]	Translation: Greek	n = 176; F: n = 136, M: n = 40 Age: mean = 71.19 ± 7.95	EWGSOP criteria: n = 50	0.96	0.96 (0.95–0.97)	SEM = 3.34 SDC = 9.24	NA, but pretest performed (n = 15)	SF-36, EQ5D Consistent construct validity (convergent and divergent)	NA	None
Yoo 2021 [27]	Translation: Korean	n = 450; F: n = 339, M: n = 101 Age: mean = 73.9 ± 6.6	EWGSOP2 criteria: n = 53	0.87	0.97 (0.97–0.98)	NA	NA, but pretest performed (n = 10)	SF-36, EQ5D Consistent construct validity (convergent and divergent)	NA	None
Beaudart 2018* [39]	Other validation (Belgium)	N = 387, F: n = 231, M: 156 Age: mean = 74.02 ± 5.99	EWGSOP1: n = 50 IWGS: n = 48 SSCWD: n = 17 FNIH: n = 38	NA	NA	NA	NA	NA	NA	NA
Geerinck 2020* [41]	Other validation (Belgium)	N = 296, F: n = 169, M: n = 127 Age: median = 73.3, range 68.9–78.6	EWGSOP criteria: n = 43 EWGSOP2 criteria: n = 13	NA	NA	NA	NA	NA	NA	NA
Geerinck 2019 [43]	Other validation (multiple countries)	n = 278; F: n = 171, M: n = 107 Age: mean = 77.67 ± 7.64	EWGSOP or FNIH criteria, all participants with sarcopenia	NA	0.97	SEM = 2.65 SDC = 7.35	NA	NA	NA	NA
Geerinck 2018 [42]	Other validation (Belgium)	N = 42, F: n = 25, M: n = 17 Age: median = 72.9, range 68.9–78.8	EWGSOP criteria, all participants with sarcopenia	NA	NA	NA	NA	NA	8 out of 9 hypotheses confirmed SRM significantly higher compared to generic instruments	NA
Witham 2022 [40]	Other validation (UK)	n = 147; F: n = 72, M: n = 75 Age: mean = 77.6 ± 7.3	EWGSOP criteria All participants with “probable sarcopenia”	0.94	NA	NA	NA	NA	MCI ranged from 5 to 21 points giving trial sample size estimates of 25–100 participant	NA

EWGSOP = European Working Group on Sarcopenia in Older People, SEM = Standard Error of Measurement, SDC = smallest detectable change, NA = not assessed, MCI = Minimal Clinical Improvement, M = Men, F = Female, CVR = Content validity ratio, CVI = Content Validity Index, FNIH Foundation for the National Institutes of Health Biomakers Consortium Sarcopenia Project, SRM = standardized response mean. *For those two specific studies, only the discriminative power of SarQoL was measured according to different diagnosis criteria of sarcopenia. The table could therefore not be completed. ‡For this specific study, SEM and SDC analysis was only performed on 12 individuals with sarcopenia, in each of the study having performed a pre-test of SarQoL, the purpose of the pre-test was to ensure the good understandability of each question, which is one of the parameters of the content validity of a scale.

### Capacity of SarQoL to detect difference in HRQoL between individuals with and without sarcopenia

**Fig. 1:**
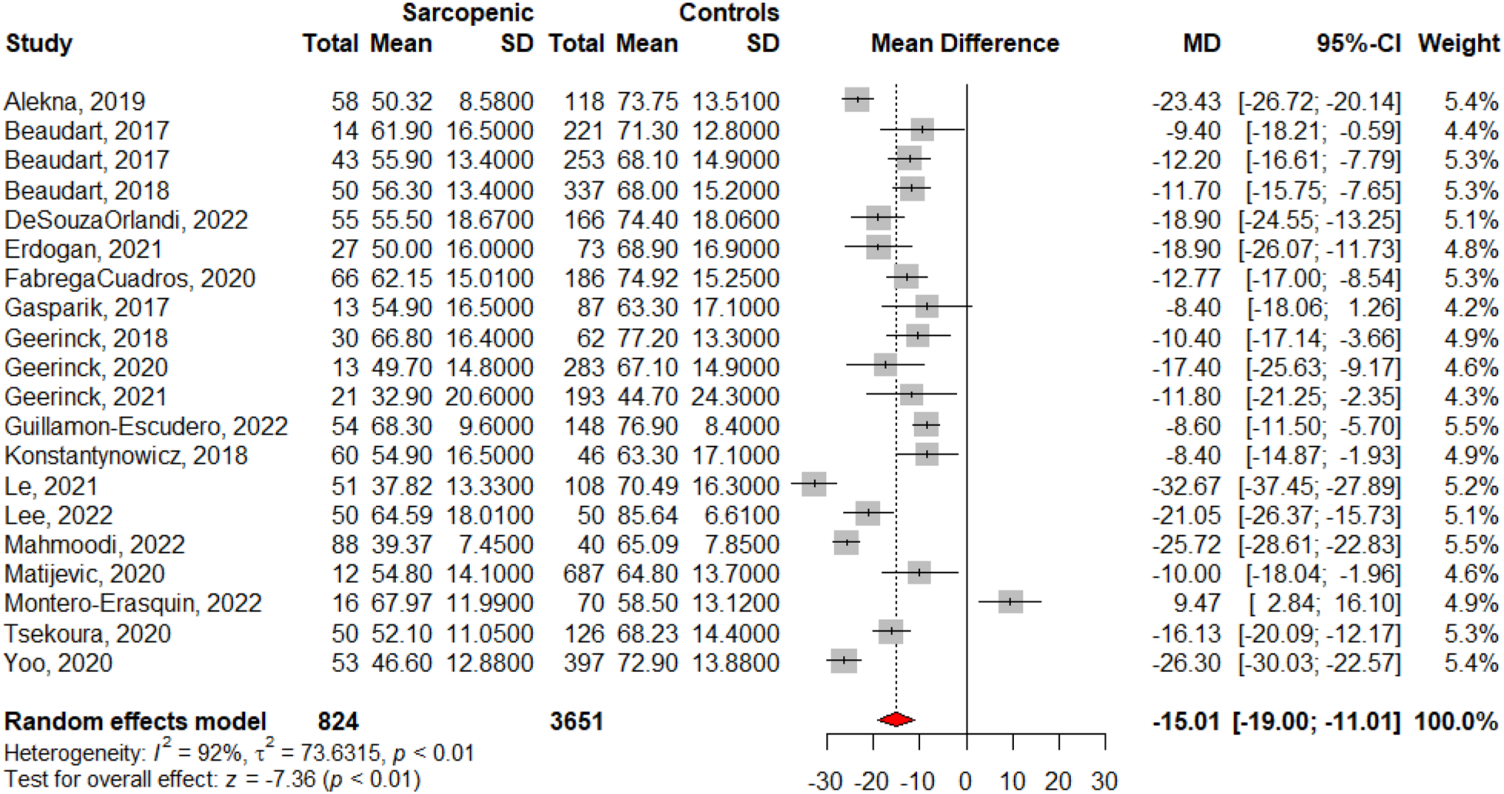
Discriminative power of SarQoL – data reported from 20 individual studies identified from a systematic literature review (Figure issued from Beaudart et al. 2023 [45])

As SarQoL is an instrument specifically designed for use in populations with sarcopenia, the ability of the questionnaire to discriminate HRQoL between individuals with and without sarcopenia must be considered. A systematic review of the literature identified 20 individual cross-sectional studies that used SarQoL to measure the quality of life in individuals with sarcopenia, diagnosed according to a consensually accepted definition, compared to individuals without sarcopenia and published until December 2022 [45]. Pooled results of these 20 individual studies using meta-analytic statistics (random effect model) showed a lower HRQoL in individuals with sarcopenia [mean difference of −15.01 points/100 (95%CI of −19.00; −11.01)] compared to individuals without sarcopenia (Fig. 1). The discriminant validity of SarQoL in regards of HRQoL using different diagnostic criteria for sarcopenia was further confirmed in two individual studies [39, 41].

### Reliability

**Internal consistency:** All 19 different validated versions of SarQoL showed an internal consistency that was considered excellent (Cronbach’s alpha > 0.8). Most validation studies also measured the effect of removing one domain at a time on global internal consistency. This statistical analysis allows the identification of a potential domain that could introduce some heterogeneity into the scale. None of the studies reported a significant change in internal consistency when a domain was removed from the total score.

**Test–retest reliability:** As SarQoL is a self-administered questionnaire, only test–retest reliability was measured and not inter-rater reliability. Almost all versions (except Romanian, Hungarian and Serbian) provided a measure of reliability. Intraclass coefficient correlations (ICCs), which are expected to be higher than 0.7 to reflect acceptable reliability of the tool, ranged from 0.93 for the Russian [34] version to 0.99 for the Ukrainian [38], Chinese [22], Persian [29], Spanish [34] and Polish [30] versions, reflecting high reliability of SarQoL, whatever the version used.

**Standard error of measurement (SEM):** SEM is a parameter used to indicate the amount of measurement error in an instrument and is consequently an indicator of reliability. Pooling together data from 9 different cohorts (278 individuals with sarcopenia) reporting values of test–retest reliability, an SEM of 2.65 points (on a scale of 0–100 points) was reported by Geerinck et al. in 2019 [43]. This value means that one can be 68% confident (± 1 SEM) that the ‘true’ score of a subject can be found between −2.65 and + 2.65 points from the observed score. The lowest SEM value was found in the Lithuanian validation study [28] (i.e., SEM = 0.18) and the highest SEM value was found in the English validation study [24] (i.e., SEM = 4.2).

**Smallest detectable change (SDC):** The SDC is defined as the change in the instrument’s score beyond measurement error and depends on the SEM. In the aforementioned publication of Geerinck et al. 2019 [43], pooling together data from 9 individual validation studies, an SDC of 7.35 (on a scale 0–100) was found. This value means that the overall HRQoL score of an individual would have to change with at least 7.35 points before the observed change can be considered to be a true change in the HRQoL of an individual, and not potentially a result of measurement error. The lowest SDC value was found in the Lithuanian validation study [28] (i.e., SDC = 0.49) and the highest SDC value was found in the English validation study [24] (i.e., SDC = 11.65).

### Validity

**Content validity:** According to the COSMIN guidelines for the assessment of content validity published in 2016 [46], two steps should be taken to measure the content validity of a scale. For the first step, it is required to evaluate the quality of PROM development, which shows that a sample of the target population was involved in the development of the items of the scale, but also that a pre-test of the scale was conducted to measure the comprehensibility and comprehensiveness of the scale. Both requirements were met during the development phase of SarQoL. Indeed, during the development of SarQoL questionnaire [13], a sample of 5 individuals with sarcopenia was involved in a qualitative interview to develop concepts and items to be included in the questionnaire. SarQoL team also sought the opinion of experts on the items that should be included in the scale. A list of 180 potential items was generated from the literature review and experts and patients’ interviews. Experts and individuals with sarcopenia were then asked to review this list and select the items they considered most relevant. Once SarQoL questionnaire was developed, a pre-test was conducted with 21 individuals to ensure a good understanding of all questions. For the second step, the COSMIN guidelines [46] require to evaluate the quality of content validity studies using the PROM by asking patients and professionals about the relevance of each item included in the scale but also by asking patients and professionals about the comprehensibility and comprehensiveness of the questionnaire. To date, only one study (i.e., Mahmoodi et al., Persian translation of SarQoL [29]) has conducted a content validity analysis according to the COSMIN guideline. The authors asked professionals about the relevance of the scale and reported a content validity ratio of 0.8–1 depending on the questions of SarQoL and a content validity index of 1, which are higher than values indicating adequate and acceptable content validity. However, patients were not interviewed in this study, and the content validity of the Persian SarQoL, as defined by the COSMIN guidelines, was therefore not entirely confirmed.

## Construct validity

Construct validity can be measured through convergent and divergent validity. All 19 validation studies compared SarQoL with the generic instruments SF-36 and EQ-5D. Hypotheses were made about the correlation between SarQoL, or some of its specific domains, with the subdomains of the SF-36 questionnaire and the EQ5D instrument that were expected to have a similar (i.e., convergent validity) or different (i.e., divergent validity) construct to SarQoL. The validity of an instrument is usually reported when more than 75% of the pre-defined hypotheses are confirmed [47]. Except for the Greek [25], Russian [32] and Ukrainian [38] versions of SarQoL, where lower-than-expected correlations were obtained, all other publications highlighted a consistent construct validity of SarQoL questionnaire for measuring HRQoL in sarcopenia. In most studies, SarQoL correlated well (r > 0.5) with the physical functioning, role limitation due to physical problems, bodily pain, general health, and vitality domains of the SF-36 questionnaire. Lower correlations (r < 0.5) were generally found with mental health and role limitation due to mental health problems. For the EQ-5D, high correlations were found with the mobility and usual activities subscales. Lower correlations were generally found with the other domains (i.e., self-care, pain/discomfort, anxiety/depression).

### Responsiveness

The responsiveness of SarQoL has been reported in two different prospective studies [38, 40]. In one of these publications, Geerinck et al. compared the specific SarQoL questionnaire with generic tools (i.e., SF-36 and EQ-5D) to detect change over time in a population of individuals with sarcopenia (n = 42). Good responsiveness was observed, as authors confirmed eight out of the nine hypotheses developed a priori, which is well above the75% confirmation threshold [48]. The standardised response mean of the total SarQoL score was significantly higher than that of the SF-36 Physical Component Summary, the EQ-5D Utility Index and the EuroQol visual analogue scale. The second publication, by Witham et al., aimed to assess the responsiveness of SarQoL in a population of individuals with probable sarcopenia (n = 147) and the suitability of SarQoL as an outcome measure in clinical trials. Using an anchor-based method, the authors reported a minimum clinical improvement after six months of follow-up that ranged from 5 to 21 points, giving trial sample size estimates of 25–100 participants, demonstrating that SarQoL is sufficiently responsive for use in clinical trials in sarcopenia. The authors also mentioned that the responsiveness of SarQoL may allow smaller sample sizes to be used in trials than implicated by use of some generic tools (for example, the EQ-5D typically requires sample sizes of 200–300 to detect the minimum clinically important difference of 0.074 points).

To date, the responsiveness of SarQoL following an intervention aimed at improving key parameters of sarcopenia (i.e., muscle mass, muscle strength, physical performance) has not been reported. According to a systematic review of the literature, eight interventional studies aimed at the treatment (pharmacological or non-pharmacological) of sarcopenia reported a measure of HRQoL as a secondary outcome [49–56]. Of these eight trials, only one used SarQoL questionnaire [49]. In this study, Tsekoura et al. [49] proposed an 12-week exercise programme to improve sarcopenia. Three groups were defined: home-based exercise, supervised exercise, and control. The results showed a significant group x time interaction for quality of life. Both exercise interventions improved HRQoL compared to the control group (supervised-exercise group + 7.28 points, home-based exercise group + 3.41 points and control group -2.19 points after 12 weeks, p < 0.05). Exercise interventions also improved physical performance and muscle strength compared to the control group. Although this study was not designed to measure responsiveness to change of SarQoL, the improvement of HRQoL and sarcopenia parameters by the intervention may be considered as an indication of the sensitivity to change of SarQoL.

### Floor & ceiling effects

No floor nor ceiling effects were reported in the 19 translation validation publications. None of the 3657 participants in the 19 language translation studies achieved the maximum or minimum score on SarQoL questionnaire.

## Short form SarQol

While the original developers of SarQoL estimated, based on the results of a pre-test in the target population, that most people would take approximately 10 min to complete SarQoL, in practice a significant number of respondents take longer. This was further confirmed in the study by Witham et al., who reported that in people with more functional limitations, the completion might take longer. Given that most clinical trials involve many tests and questionnaires, it seems worth considering whether it would be possible to reduce the related burden on trial participants by reducing the size of the questionnaire. With this intention, Geerinck et al. developed a shorter version of SarQoL questionnaire in 2021, hereafter referred to as SF-SarQoL [57]. Following a two-stage item reduction process, the full SarQoL was reduced from 55 to 14 items (i.e., a 75% reduction). The authors investigated the clinimetric properties of this new version and confirmed that equivalence was achieved. Indeed, SF-SarQoL discriminated well between participants with and without sarcopenia, had an excellent internal consistency (α = 0.915, ω = 0.917) and an excellent test–retest reliability (ICC = 0.912 [0.847–0.942]). For this new format of the questionnaire, the authors also investigated the structural validity of the questionnaire and examined the item parameters with a graded response model (IRT). As a result, an unidimensional model was fitted with no misfitting items and a good response category.

## Discussion / expert commentary

Currently, SarQoL is the only specific HRQoL questionnaire for individuals with sarcopenia available in the literature. Another sarcopenia-specific PROM, the Age-Related Muscle Loss Questionnaire (ARMLQ), was developed in 2011 [58]. Even if both SarQoL and ARMLQ provide information on the patient’s perspective, only SarQoL evaluates HRQoL, while the ARMLQ restricts its domains of interest to the functional impact of reduced muscle strength. Moreover, the psychometric performances of the ARMLQ have not yet been reported.

The current review presents an overview of all the psychometric properties of SarQoL measured in different translation and validation studies. It is important to note that most translated versions of SarQoL showed similar psychometric properties. Demonstrating that a tool is consistently valid and reliable in different populations from different countries using different diagnostic criteria for sarcopenia makes the available evidence robust.

In addition, a recent systematic review that aimed to analyse the structural characteristics and psychometric properties of translated versions of SarQoL questionnaire supports the conclusion of the current review. After carefully assessing the psychometric properties of the translated versions of SarQoL based on the COSMIN guidelines [20, 59], the authors concluded that the analysed versions have psychometric properties that can be qualitatively classified between good and excellent. They state that SarQoL is valid for assessing the quality of life in people with sarcopenia in different countries [44]. The authors however regret the absence of content validity measurement across the different validation analyses. Since 2021, SarQoL has been recognised by the European Society for Clinical and Economic Aspects of Osteoporosis, Osteoarthritis and Musculoskeletal Diseases (ESCEO), as the official tool for measuring HRQoL in sarcopenia. Furthermore, the recent revision of EWGSOP2 recommends using SarQoL questionnaire in clinical care and research studies [4].

Despite the available evidence of the suitability of SarQoL to measure HRQoL for individuals with sarcopenia, research on the psychometric properties of SarQoL questionnaire should continue.

- First, the responsiveness to change of SarQoL has still not been measured in the context of interventional studies. It is therefore still uncertain if SarQoL may be sensitive enough to detect HRQoL changes associated with improvements in muscle mass or muscle strength following a pharmaceutical or non-pharmaceutical intervention. The only clinical trial that used the SarQoL questionnaire as a secondary outcome showed an improvement of HRQoL following a non-pharmaceutical intervention, which may suggest that SarQoL may be sensitive to change [49]. However, this is currently only an assumption based on a small amount of preliminary evidence, and no statistical measure of the sensitivity to change using anchor questions was performed.
- Second, as highlighted in the systematic review by Martinez-Fernandez [44], the content validity of SarQoL has rarely been studied so far. Because content validity is a psychometric property recognized by COSMIN, future studies should investigate this property in different populations. Currently, researchers interested in the translation and validation of SarQoL in another language are requested to follow the guidance provided by the developers of SarQoL. This guidance has now been updated to include a per se measurement of the content validity. It is now recommended to conduct patient interviews or focus groups using open-ended methods to elicit patients’ input. Additional evidence regarding this psychometric property should therefore be available in the next few months.
- Third, most of the available evidence summarized in this review was obtained from cross-sectional studies. Very few longitudinal studies have provided data on the evolution of HRQoL in individuals with sarcopenia. We encourage the use of SarQoL in prospective studies to provide new evidence on the impact of sarcopenia on HRQoL and strengthen the sensitivity to change analyses.
- Fourth, currently, no cut-off score exists to define a low HRQoL for sarcopenia. The score of SarQoL is currently only used as a continuous value. Providing a cut-off for the definition of a low HRQoL is scientifically challenging. Nevertheless, it may be relevant to develop such a cut-off to identify individuals with sarcopenia with a particularly low HRQoL in which specific health action targeting HRQoL could be proposed.
- Fifth, SarQoL has only been used in community-dwelling older individuals with sarcopenia and has never been studied in populations such as those living in nursing homes or those with severe cognitive impairment. In order to extend the applicability of SarQoL to other population profiles, it may be interesting to test the psychometric properties of this questionnaire in these different populations as well.

## Conclusion

Although studies are underway or planned to further characterise the psychometric properties of SarQoL, this literature review shows that SarQoL can be used in observational and interventional studies to validly assess sarcopenia-specific HRQoL in older individuals with sarcopenia. Disease-specific instruments such as SarQoL should be used to complement measurements from generic questionnaire, as generic measures are still essential to assess broader health status in older people who usually suffer from multiple conditions.

## Supplementary Information

Below is the link to the electronic supplementary material.

Supplementary file1 (PDF 630 KB)

## Data Availability

NA.
