# Supplementary material for: Measuring health-related quality of life in sarcopenia: summary of the SarQoL psychometric properties
Source: Aging Clin Exp Res. 2023 May 23;35(8):1581–93. doi: 10.1007/s40520-023-02438-3 (PMC10363087; doi:10.1007/s40520-023-02438-3)
Supplement: Supplementary file 1 — Supplementary file1 (PDF 630 KB) [file 40520_2023_2438_MOESM1_ESM.pdf]

SarQoL
Sarcopenia and Quality of Life

Quality of life in sarcopenia

This questionnaire asks about sarcopenia, which is a muscle weakness that comes about with ageing. Sarcopenia can affect your daily life. This survey will enable us to find out if the state of your muscles currently affects your quality of life.

Please choose the most appropriate response for each question. The questionnaire should take you approximately 10 minutes to complete.

1. Do you currently feel you have a reduction in:

	A lot	Some	A little	None
The strength in your arms?	☐	☐	☐	☐
The strength in your legs?	☐	☐	☐	☐
Your muscle mass?	☐	☐	☐	☐
Your energy?	☐	☐	☐	☐
Your physical capabilities?	☐	☐	☐	☐
Your general flexibility?	☐	☐	☐	☐

2. Do you have pain in your muscles?

- ☐ Often
- ☐ Sometimes
- ☐ Rarely
- ☐ Never

Copyright© - January 2015 - All right Reserved

3. When undertaking light physical activities (walking slowly, doing the ironing, dusting, washing-up, DIY, watering the garden, etc.), do you:

	Often	Occasionally	Rarely	Never	I do not undertake these types of physical activities
Have difficulty?	☐	☐	☐	☐	☐
Get tired?	☐	☐	☐	☐	☐
Experience pain?	☐	☐	☐	☐	☐

4. When undertaking moderate physical activities (fast walking, cleaning windows, hoovering, washing the car, pulling up weeds in the garden, etc.), do you:

	Often	Occasionally	Rarely	Never	I do not undertake these types of physical activities
Have difficulty?	☐	☐	☐	☐	☐
Get tired?	☐	☐	☐	☐	☐
Experience pain?	☐	☐	☐	☐	☐

5. When undertaking intense physical activities (running, hiking, lifting heavy objects, moving furniture, digging the garden, etc.), do you:

	Often	Occasionally	Rarely	Never	I do not undertake these types of physical activities
Have difficulty?	☐	☐	☐	☐	☐
Get tired?	☐	☐	☐	☐	☐
Experience pain?	☐	☐	☐	☐	☐

6. Do you currently feel old?

- ☐ Yes, very
- ☐ Yes, somewhat
- ☐ Yes, a little
- ☐ No, not at all

Copyright© - January 2015 - All right Reserved

7. If yes to question 6, what gives you that impression?

(Choose as many answers as you like)

- ☐ I become unwell easily
- ☐ I take many medications
- ☐ I feel a weakness in my muscles
- ☐ I have problems with my memory
- ☐ I've had to face the death of several people close to me
- ☐ I do not have much energy, I am often tired
- ☐ My eyesight is poor
- ☐ Other:

8. Do you feel physically weak?

- ☐ Yes, completely
- ☐ Yes, somewhat
- ☐ Yes, a little
- ☐ No, not at all

9. Do you feel you are limited in:

	A lot	Some	A little	None
The length of time you can walk for?	☐	☐	☐	☐
How often you go out walking?	☐	☐	☐	☐
The distance you can walk?	☐	☐	☐	☐
The speed at which you can walk?	☐	☐	☐	☐
The length of your steps?	☐	☐	☐	☐

10. When you are walking:

	Often	Occasionally	Rarely	Never	I am unable to walk
Do you feel very tired?	☐	☐	☐	☐	☐
Do you need to sit down regularly to recover?	☐	☐	☐	☐	☐
Do you have difficulty crossing roads quickly enough?	☐	☐	☐	☐	☐
Do you have difficulties with uneven surfaces?	☐	☐	☐	☐	☐

Copyright© - January 2015 - All right Reserved

11. Do you have problems with your balance?

- ☐ Often
- ☐ Occasionally
- ☐ Rarely
- ☐ Never

12. How often do you fall?

- ☐ Very often
- ☐ Occasionally
- ☐ Rarely
- ☐ Never

13. Do you think that your physical appearance has changed?

- ☐ Yes, very
- ☐ Yes, somewhat
- ☐ Yes, a little
- ☐ No, not at all

14. If yes to question 13, in what way? (Choose as many answers as you like)

- ☐ Change in your weight (you've put on weight or you've lost weight)
- ☐ Appearance of wrinkles
- ☐ Loss of height
- ☐ Loss of muscle mass
- ☐ Hair loss
- ☐ Getting white or grey hair
- ☐ Other:

15. If yes to question 13, are you upset by this change?

- ☐ Yes, very
- ☐ Yes, somewhat
- ☐ Yes, a little
- ☐ No, not at all

Copyright© - January 2015 - All right Reserved

16. Do you feel frail?

- ☐ Very much so
- ☐ A little
- ☐ Not at all

17. Do you currently have difficulty in undertaking any of the following daily activities:

	Unable to do	Great difficulty	A little difficulty	No difficulty	Not applicable
Climbing a flight of stairs?	☐	☐	☐	☐	☐
Climbing several flights of stairs?	☐	☐	☐	☐	☐
Going up one or several steps without holding on to the banister?	☐	☐	☐	☐	☐
Squatting or kneeling?	☐	☐	☐	☐	☐
Stooping or leaning down to pick up an object off the floor?	☐	☐	☐	☐	☐
Getting up from the floor without holding on to anything?	☐	☐	☐	☐	☐
Getting out of a low chair without armrests?	☐	☐	☐	☐	☐
Moving, generally, from a sitting position to a standing position?	☐	☐	☐	☐	☐
Carrying heavy objects (large bags full of shopping, saucepan filled with water, etc.)?	☐	☐	☐	☐	☐
Opening a bottle or a jar?	☐	☐	☐	☐	☐
Using public transport?	☐	☐	☐	☐	☐
Getting in or out of a car?	☐	☐	☐	☐	☐
Doing your shopping?	☐	☐	☐	☐	☐
Doing the housework (making the bed, hoovering, doing the ironing, washing the dishes, etc.)?	☐	☐	☐	☐	☐

Copyright© - January 2015 - All right Reserved

18. Does your muscle weakness limit your movement?

- ☐ Yes, a lot
- ☐ Yes, somewhat
- ☐ Yes, a little
- ☐ No, not at all

19. If yes to question 18, for what reasons? (Choose as many answers as you like)

- ☐ Fear of pain
- ☐ Fear that you might not be able to
- ☐ Fear of feeling tired after these activities
- ☐ Fear of falling
- ☐ Other:

20. Does your muscle weakness limit your sex life?

- ☐ I am not sexually active
- ☐ Yes, completely
- ☐ Yes, somewhat
- ☐ Yes, a little
- ☐ No, not at all

21. How has your participation in physical activities/sport changed?

- ☐ Increased
- ☐ Decreased
- ☐ Unchanged
- ☐ I have never participated in physical activities or sports

22. How has your participation in leisure activities (going out to eat, gardening, doing DIY, shooting/fishing, senior citizens clubs, playing bridge, going for a walk, etc.) changed?

- ☐ Increased
- ☐ Decreased
- ☐ Unchanged
- ☐ I have never participated in leisure activities

Copyright© - January 2015 - All right Reserved
